# Supplementary material for: Separable EEG Features Induced by Timing Prediction for Active Brain-Computer Interfaces
Source: Sensors (Basel). 2020 Jun 25;20(12):3588. doi: 10.3390/s20123588 (PMC7348905; doi:10.3390/s20123588)
Supplement: Supplementary file 1 [file sensors-20-03588-s001.pdf]

Supplementary materials

S1: behavioral performances of training session

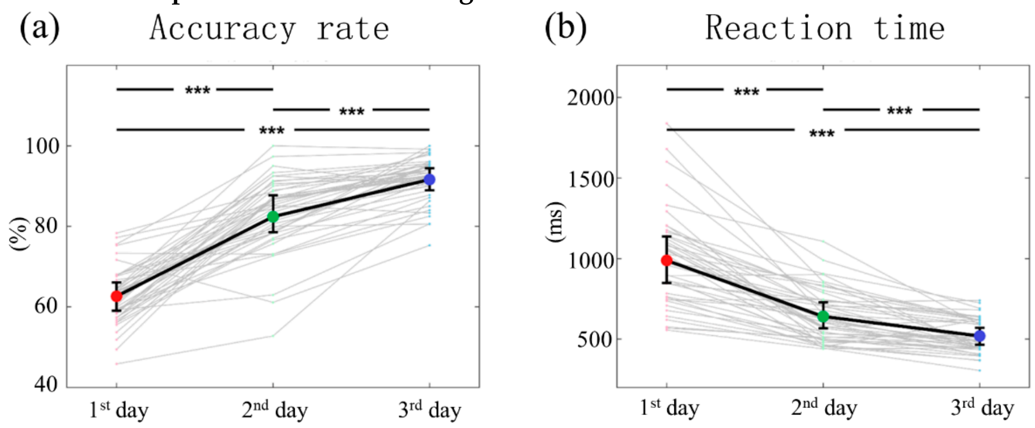

sFigure1 (a) The response accuracies of different training days. The small dots indicate the accuracy of each subject (totally 45 subjects), while the big dots indicate the averaged value. (b) The reaction times of different training days. Statistical significances: * 0.01<P<0.05; ** 0.001<P<0.01; ***P<0.001 (Post hoc tests after Bonferroni correction). This figure could also be found in https://doi.org/10.1007/s12264-020-00527-1.

Obviously, with the increasement of training days, subjects had a significant ascending trend for response accuracy ($F(2, 88)$ =286.124; P<0.001) but a significant descending trend for reaction time ($F(2, 88)$ =116.453; P<0.001). To be specific, (sFig.1b) the accuracy of the third day (92.36%) was significantly higher than those of the second (83.39%, P<0.001 after Bonferroni correction) and the first day (62.58%, P<0.001 after Bonferroni correction), and the accuracy of second day significantly higher than that of the first day (P<0.001 after Bonferroni correction). The reaction time was also significantly reduced by training ($F(2, 88)$ =287.121; P<0.001). Specifically, the reaction time was the shortest for the third day (506.15ms), medium for the second (638.46ms) and longest for the first day (1009.17ms), as displayed in sFig.1b. It demonstrates the effectiveness of trainings for the subjects on the constructing the idea about the three timing intervals in their minds.

Notably, as the ability of perceiving timing intervals varied with subjects, so the training blocks for each subject were different. In each training day, the subjects with better time perception, only did about 5 training blocks; whereas the ones who was insensitive to perceive the timing intervals, needed to train for 10~20 blocks. For this reason, the current study mainly calculated the averaged behavioral result of each training day. As to the sentence "at least three training blocks successively", it was monitored by the experimenter during training.

S2: lateralization potentials for measuring the influence of button-press

Allowing for the subjects needed to make judgement by pressing button, it remains unclear whether it could influence the neural signatures of timing prediction or not. Therefore, we calculated the lateralization potentials to measure the influence of button-press on the ERP profiles. After aligning the data to the button-press moment (zero point in sFigure.2(a)), the lateralization potential of typical motor-related electrodes (C3, C4) was calculated as follows:

$$AMP_{c3c4dif} = (AMP_{c3con} - AMP_{c3ips}) + (AMP_{c4con} - AMP_{c4ips})$$

Where $AMP_{c3c4dif}$ represents lateralization potential, AMP_{c3con} represents the amplitude in C3 electrode when subjects pressed button using right hand (contralateral), while AMP_{c3ips} represents amplitude in C3 electrode when subjects pressed button using left hand (ipsilateral); so did C4. When the lateralization potential is zero, it means movement does not have an influence on ERP profiles, whereas the larger its value is, more obvious the its influence is.

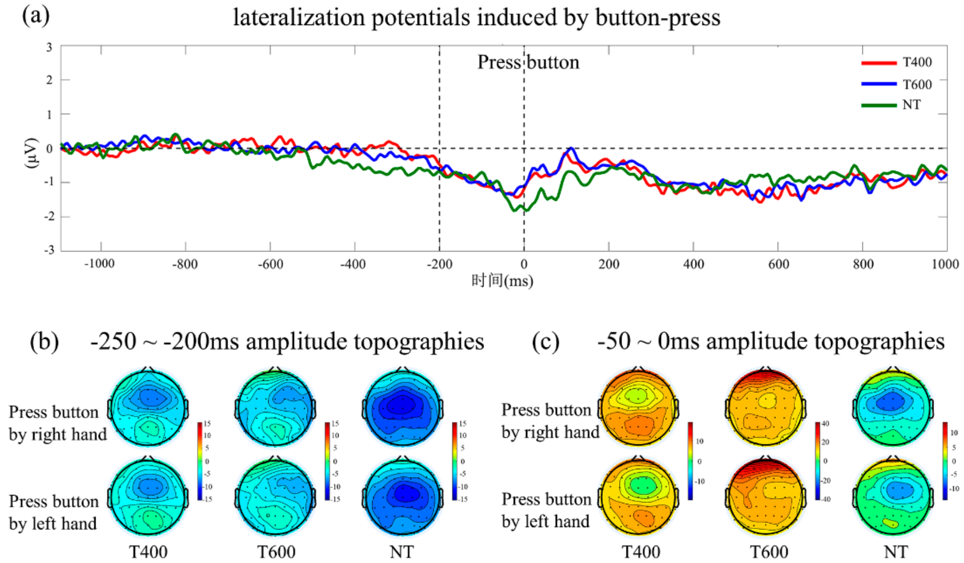

sFigure2 (a) lateralization potentials induced by button-press. (b) -250~-200ms; (c) -50~0ms amplitude topographies when subjects pressed the button using right (upper) or left (bottom) hand.

sFigure.2(a) showed the lateralization potentials induced by button-press. Obviously, in T400 and T600 conditions, the lateralization potentials emerged at about 200ms before button-press, while that of NT emerged earlier. It is reasonable for the NT to result in earlier lateralization potential, as the subjects constantly pressed the button by only one hand in whole experimental block. sFigure 2(b) and 2(c) were the amplitude topographies covering -250~-200ms and -50~0ms relative to the button-press moment. It is evident that the former period was rarely influenced by button-press, whereas the later period was influenced. These observations indicated, in this study, the influence of movement was mainly within 200ms before button-press. Thus, during the period that we selected for classification, ERP separations between T400 and T600 is mainly attributed to timing prediction, rather than button-press.

S3: combined DCPM and CSP method is more suitable for current EEG features

In future studies, we may be able to find better classification methods. However, we think the current method, i.e., the combined DCPM and CSP method, is more suitable for the EEG features in this study. In supplementary materials, we compared the current methods with part of other machine learning methods in four aspects. Firstly, we compared the effects of classifier. In the current study, after the feature extraction, Fisher discriminative analysis (FDA) is used for distinguishing the T400 and T600 signals, we argue that the effect of FDA is similar to other classifiers, such as the support vector machine (SVM). In supplementary materials sTable1, the classification accuracies of DC+PM, DC+FDA, DC+SVM were compared. It should be noted that the DCPM method contains both feature extraction (DSP filter and CCA method, I.e., DC)

and classification (Pattern Matching, PM) procedure. After the 'DC' feature extraction, there were three methods used for classification, i.e., PM, FDA and SVM.

sTable 1 classification accuracy based on 0~3Hz data.

Subject	DC+PM	DC+FDA	DC+SVM
1	67.74	66.13	59.23
2	56.25	63.75	68.75
3	68.75	65.00	68.75
4	56.25	68.75	67.50
5	75.00	76.25	80.00
6	62.50	55.00	63.75
7	62.50	61.25	66.25
8	81.25	73.75	73.75
9	73.75	71.25	63.75
10	57.50	65.00	67.50
11	60.00	66.25	65.00
12	72.50	70.00	70.00
13	55.00	60.00	55.00
14	72.50	68.75	55.00
15	66.25	60.00	66.25
16	72.50	73.75	70.00
17	53.50	50.00	51.25
18	60.25	58.50	66.25
Mean	65.22	65.19	65.45
Std	7.93	6.70	6.74

Obviously, different classifiers almost did not influence the classification performances of both features in time (sTable1) and frequency (sTable2) domain.

sTable 2 classification accuracy based on 20~60Hz data.

Subject	CSP+FDA	CSP+SVM
1	80.65	80.90
2	68.75	71.25
3	58.75	60.00
4	88.75	89.00
5	62.50	65.00
6	60.00	57.50
7	68.75	70.50
8	56.25	60.00
9	70.00	66.25
10	53.75	50.00
11	70.00	68.75
12	92.50	92.50
13	71.25	75.00
14	71.25	73.75
15	81.25	77.50
16	90.00	91.50
17	70.00	72.50
18	62.50	62.50

Mean	70.94	71.36
Std	11.26	11.48

sTable 3 classification accuracy based on 0~3Hz data.

Subject	LDA	SWLDA	DCPM
1	61.57	63.57	67.74
2	56.25	59.25	56.25
3	59.75	61.75	68.75
4	63.75	62.77	56.25
5	62.50	63.50	75.00
6	51.25	54.62	62.50
7	52.50	51.70	62.50
8	52.50	62.50	81.25
9	55.00	58.00	73.75
10	56.25	58.75	57.50
11	53.75	54.99	60.00
12	67.50	67.50	72.50
13	52.50	51.50	55.00
14	62.50	60.40	72.50
15	63.75	69.75	66.25
16	55.00	59.00	72.50
17	56.25	55.75	53.50
18	55.50	58.50	60.25
Mean	57.67	59.66	65.22
Std	4.71	4.80	7.93

sTable 4 accuracy comparison.

Subject	DCPM+CSP Decision level	DCPM+CSP Feature level
1	70.97	80.65
2	72.50	71.25
3	68.75	68.75
4	92.50	88.75
5	86.25	80.00
6	57.50	53.75
7	71.25	68.75
8	78.75	75.00
9	75.00	71.25
10	72.50	70.00
11	78.75	81.25
12	88.75	92.50
13	70.00	68.75
14	67.50	75.00
15	85.00	87.50
16	87.50	87.50
17	71.25	72.50
18	60.00	56.25
Mean	75.26	74.97
Std	16.25	16.18

Secondly, we compared the DCPM performance with traditional classification methods, such as linear discriminative analysis (LDA), stepwise LDA (SWLDA), (the details about LDA and SWLDA please see the reference named ‘Discriminative canonical pattern matching for single-trial classification of ERP components’). As shown in sTable3, DCPM achieved better performance.

Thirdly, there were two kinds of combination methods, i.e., combination in decision level and in feature (for details about the two methods, please see reference ‘enhance decoding of pre-movement EEG patterns for brain-computer interfaces’). As shown in sTable4, the two methods had almost similar performance.

Fourthly, some deep learning algorithm may have better performance, but the data set of current data was limited (40 trials at most), which obstructs the use of these algorithms. In sum, the current classification method is more suitable for the current EEG features.
